# Maxillofacial Bone Involvement in Fibro-Osseous Lesions: Emphasizing the Significance of Differential Diagnosis

**DOI:** 10.3390/jcm13113233

**Published:** 2024-05-30

**Authors:** Paolo Gennaro, Luigi Gennari, Linda Latini, Guido Cavati, Margherita Vannucchi, Filippo Giovannetti, Flavia Cascino

**Affiliations:** 1 Maxillofacial Surgery Unit, Department of Mental Health and Sense Organs, Santa Maria Le Scotte, University Hospital of Siena, Viale Mario Bracci, 16, 53100 Siena, Italy; 2 Department of Medicine Surgery and Neuroscience, University of Siena, Viale Mario Bracci, 16, 53100 Siena, Italy; 3 Section of Pathology, Department of Medical Biotechnology, University of Siena, 53100 Siena, Italy; 4 Maxillofacial Surgery, San Salvatore Hospital, Department of Life Health and Environmental Sciences, University of L’Aquila, 67100 L’Aquila, Italy

**Keywords:** fibrous dysplasia, ossifying fibroma, WHO, fibro-osseous lesion, maxillo-facial bones

## Abstract

The World Health Organization’s (WHO) 2022 update on the classification of odontogenic and maxillofacial bone tumors has revolutionized diagnostic and treatment paradigms by integrating novel molecular insights. Fibro-osseous lesions of the maxillo-facial bones constitute a heterogeneous group encompassing fibrous dysplasia, Psammomatoid Ossifying Fibroma (PSOF), Juvenile Trabecular Ossifying Fibroma (JTOF), and other variants. Despite histological similarities, their distinct clinical manifestations and prognostic implications mandate precise differentiation. The intricacies of diagnosing fibro-osseous lesions pose challenges for pathologists, maxillofacial surgeons, dentists and oral surgeons, underscoring the importance of a systematic approach to ensure optimal patient management. Herein, we present two cases, fibrous dysplasia and Cemento-Ossifying Fibroma, detailing their clinical encounters and management strategies. Both patients provided informed consent for publishing their data and images, adhering to ethical guidelines.

## 1. Introduction

In 2022, the WHO updated its classification of odontogenic and maxillo-facial bone tumors thanks to the ensuing novel molecular findings which have clinical applications and can set the stage for the beginning of a new era of treatment approaches.

The group of fibro-osseous lesions of the jawbones includes several diagnostic subtypes including fibrous dysplasia (FD), Psammomatoid Ossifying Fibroma (PSOF), Juvenil Trabecular Ossifying Fibroma (JTOF), Familial Gigantiform Fibroma, Familial Forid Cemento-Osseous Dysplasia (FamFLCD), Odontogenic Cemento-Ossifying Fibroma (COF), Cement-Osseous Dysplasia, itself subdivided into three forms, and Segmental Odonto-Maxillary Dysplasia (SOD), newly introduced in the latest classification [1].

Generally, fibro-osseous lesions have similar microscopic histological features, characterized by a fibrous stroma with various types and amounts of mineralized products [2].

The three major forms are fibrous dysplasia, ossifying fibroma, and cemento-osseous dysplasia; however, it is useful to remember that according to the latest classification, it is wrong to consider ossifying fibroma as a single nosological entity, as its various subtypes are currently recognized as separate entities.

Fibrous dysplasia is a rare disease that affects 1 in 30,000 people and accounts for 2.5% of bone lesions and 1% of all primary bone tumors [3]. It is a localized bone disorder characterized by an abnormal proliferation of fibrous tissue mixed with healthy and immature bone. It may be restricted to a single bone (monostotic form) or may involve multiple bones simultaneously (polyostotic form). It could be associated with endocrinopathies, severe bone deformities, and several clinical manifestations, including characteristic skin lesions called “coffee and milk spots”, constituting McCune Albright Syndrome [4].

Cemento-osseous dysplasia is the most common fibro-osseous lesion of the jaws; it is associated with the tooth-bearing areas of the gnathic bone, and it is characterized by a reactive process where normal bone is replaced by a poorly cellularized cementum-like material and cellular fibrous connective tissue [5].

Psammomatoid Ossifying Fibroma (PsOF), Juvenil Trabecular Ossifying Fibroma (JTOF), and Odontogenic Cemento-Ossifying Fibroma are benign bone neoplasms that affect the jaws and bones of the craniofacial region; however, they could have significant growth potential. Individually they have different features, but they share the fact that they are readily identified intraoperatively because they are well-demarcated [2].

Due to their similar features, fibro-osseous lesions are challenging not only for the pathologist but also for the maxillofacial surgeon, so a systematic approach to evaluating such cases is essential to avoid diagnostic pitfalls and to ensure the most appropriate diagnoses to guarantee a proper treatment and an informative prognosis.

## 2. Detailed Case Description

The authors present the encounter and management of two of these fibrous bone lesions, specifically fibrous dysplasia and Cemento-Ossifying Fibroma.

Both patients signed informed consent and, according to the principles of the Declaration of Helsinki, signed consent for the publication of their data and photographs.

### 2.1. Case 1

In January 2022, a 43-year-old woman came to the attention of the Maxillofacial Surgery Unit of Azienda Ospedaliera Universitaria Senese of Siena for a worsening headache of some years. On physical examination, the eurythmy of the face was preserved, and facial asymmetries were absent; moreover, neither sensory nerves nor olfactory or visual deficits were present. There were also no “coffee and milk stains” in any explored body area.

**Figure 1:**
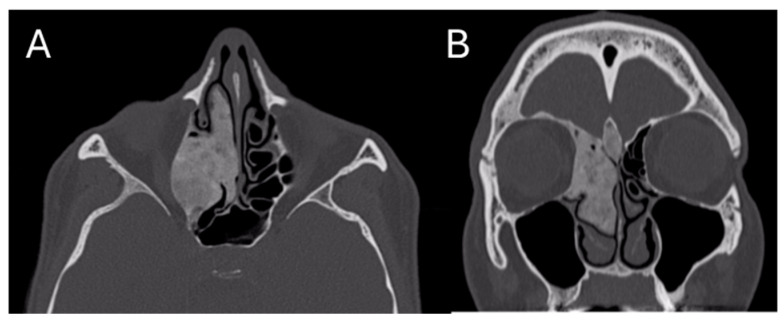
Pre-operative CT Image of Patient 1. (**A**) An axial cut; (**B**) a coronal cut. In both, the extension of the lesion into the ethmoidal region is evident.

The patient had already performed a skull CT scan that showed an osteostructural alteration with a swollen appearance of the bone component of the right ethmoidal cells, with the encumbrance of the homolateral nasal fossa and the probable involvement also of the homolateral orbital walls; the neoformation was 24 × 43 × 38 mm (Figure 1).

These findings showed an increased extension compared to a previous examination performed in 2013, executed for the same symptomatology. She also underwent head and neck Magnetic Resonance Imaging (MRI) that confirmed the presence of coano-ethmoidal neoformation which was hypointense in all the basic sequences indicating a greater fibrous-calcific composition, which did not restrict in diffusion and had an expansive aspect with orbital extension and the commitment of the homolateral optical channel. After the administration of contrast, an impregnation of the lesion was detected with extension to the olfactory showers with a prevalence on the right where it extends to the temporal pole.

In consideration of the optical canal commitment, the patient was also subjected to an eye examination with the study of the visual field through Snellen’s Test, which did not show alterations of the visus.

The patient did not present other noteworthy pathologies and did not take chronic therapies. She had regular menstrual cycles under estro-progestinic contraceptive therapy. There were no endocrine pathologies in the anamnesis.

Considering the radiological suggestive bone nature of the lesion, after a collegial discussion of the case with the medical internist, an incisional biopsy of the middle turbinate in the right nasal fossa was scheduled.

**Figure 2:**
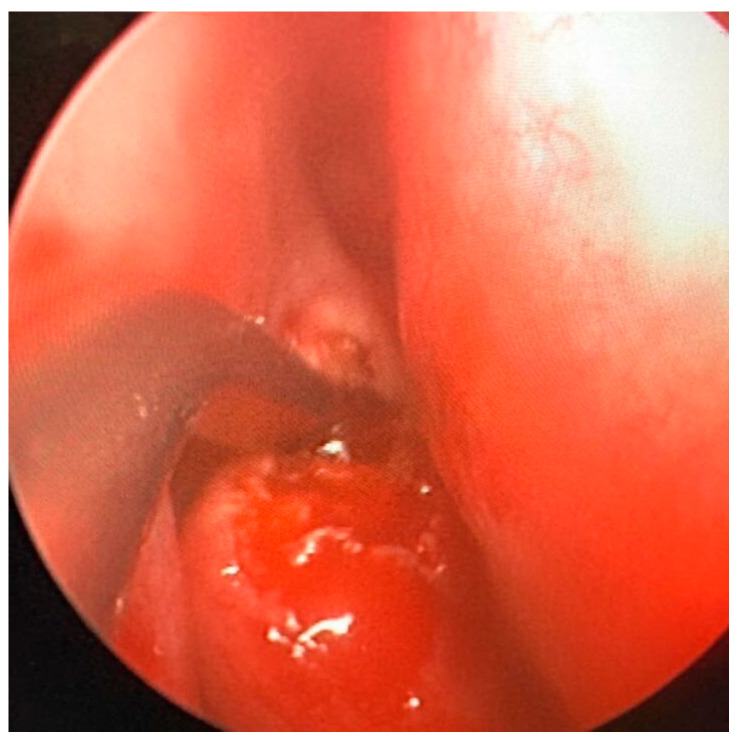
Intra-operative fragmentation of the middle turbinate. The intraoperative phase is appreciated, during which the middle turbinate was biopsied using Piezo-surgery.

Under general anesthesia, an endoscopy of the right nasal cavity was performed, and the hypertrophy of the middle turbinate was appreciated, with a normotrophic overlying mucosa. A biopsy was attempted with Weil–Blakesley forceps, but due to the bone consistency of the lesion it was preferred to perform, through Piezo-Surgery [6] a fragmentation of the medium turbinate with a subsequent sampling of two operating samples of 15 × 25 mm and 10 × 10 mm, respectively (Figure 2).

At the end of the procedure, hemostasis was performed with cauterization and the subsequent placement of two Merocel-type swabs in the right nasal fossa, which were subsequently removed on third day. No intraoperative complications were observed.

Samples were sent both to the Institutes of Pathological Anatomy of the Azienda Ospedaliera Universitaria Senese and the Institutes of Pathological Anatomy of Umberto I Hospital in Rome.

**Figure 3:**
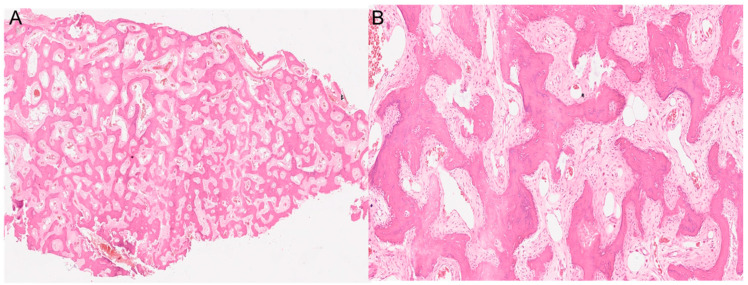
The histopathological images show a section of the middle turbinate. (**A**) 4× magnification. (**B**) 20× magnification. Both images show the main characteristics of fibrous dysplasia, such as fibrous tissue intermixed with irregularly distributed bony trabeculae.

Both histological reports confirmed the diagnosis of fibrous dysplasia, showing a bone-fibrous fragment including bone trabeculae with irregular distribution with a crossed fibers structure with marked remodeling, and a non-atypic fibrous tissue with several vascular structures with thick and mineralized walls (Figure 3).

Moreover, in continuity with this tissue, they showed a de-epithelialized mucosa with normal gland structures. The Ki67 expression shown was <5%.

Collaterally at the laboratory of the Internal Medicine and Complexity Department, the dosage of calcemia, phosphoremia and alkaline phosphatase was acquired, which were within the limits. Bone markers were also acquired, showing only mild hypovitaminosis D (25-OH Vitamin D 28.1 ng/mL v.n. 30–100 ng/mL).

Other blood chemistry tests including blood count, hepatorenal function, electrolytes, and coagulation were within the limits.

We aimed to monitor the S-CTX values to evaluate the eventual infusive therapy with antiresorptive agents.

The patient underwent clinical follow-up every three months at the maxillofacial surgery outpatient clinics for the two years following the intervention. Throughout this period, no signs of worsening were reported.

We also scheduled periodical CT control to value any infiltration of the optic canal and a periodic eye examination to highlight any abrupt visual impairments, to be able to act promptly.

### 2.2. Case 2

**Figure 4:**
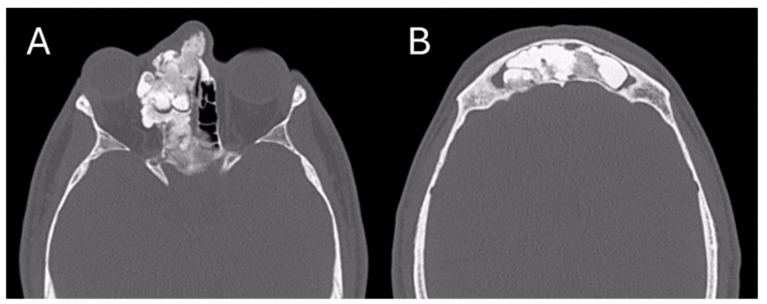
Pre-operative CT image of Patient 2. Two axial cuts are appreciated (**A**) The lesion localized at the ethmoidal level (**B**) The lesion localizedin the right frontal sinus.

In July 2023, a 35-year-old man came to the attention of the Maxillofacial Surgery Unit of Azienda Ospedaliera Universitaria Senese of Siena to evaluate a facial asymmetry that worsened over the years. On physical examination, the eurythmy of the face was not preserved with marked exophthalmos and a dystopia of the right eye; the patient reported no sensory nerve abnormalities nor olfactory or visual deficits. The patient also reported a history of sinusitis. The skull CT scan was already performed, and it showed a large calcific-fibrotic-mixed component neoformation centered in the body of the ethmoid, extending caudally towards the right nasal fossa and cranially towards the frontal sinus occupying it bilaterally. Posteriorly, the lesion extended towards the sphenoid projecting into the anterior cranial fossa, anteriorly, and laterally towards the right orbit (Figure 4).

An incisional biopsy of the middle turbinate in the right nasal fossa was performed. Under general anesthesia, an endoscopy of the right nasal cavity was performed, and a hypertrophy of the middle turbinate was appreciated, with a normotrophic overlying mucosa. A biopsy was attempted with Weil–Blakesley forceps, and one operating sample was sent to the Institute of Pathological Anatomy of the Azienda Ospedaliera Universitaria Senese. At the end of the procedure, hemostasis was performed with cauterization and the subsequent placement of one Merocel-type swab in the right nasal fossa, subsequently removed on the third day. No intraoperative complications were observed. The histological biopsy report supported the diagnosis of ossifying fibroma, which was not otherwise specified.

Collaterally, at the laboratory of the Internal Medicine and Complexity Department, the dosage of calcemia, phosphoremia and alkaline phosphatase was acquired, which were within the limits (Ca 8.7 mg/dl v.n. 8.6–10.4 mg/dl, P 3.8 mg/dl v.n. 2.5–4.8 mg/dl, ALP 75 U/L v.n. 30–120 U/L).

One month after the biopsy, the patient was admitted to the emergency room in Siena due to a sudden edema in the right periorbital area following a sneeze. Therefore, given the histological diagnosis obtained and the patient’s clinic, which suggested a possible worsening due to the increase in the size of the neoformation and the increasing fragility of the bone segments, it was decided to schedule surgery to completely remove the lesion using a combined maxillofacial and neurosurgical approach.

In the first maxillofacial time, coronal access was performed by a pretrichial “crown” incision [7]. The pericranial flap was harvested and the frontal bone was exposed up to the upper orbital frames bilaterally to show the bone lesions at the level of the naso-glabellar suture.

After the neurosurgeon performed a bilateral craniotomy, the maxillofacial surgeon performed a Raveh-type osteotomy [8] by which the ossifying neoformation in the frontal sinus was exposed bilaterally.

**Figure 5:**
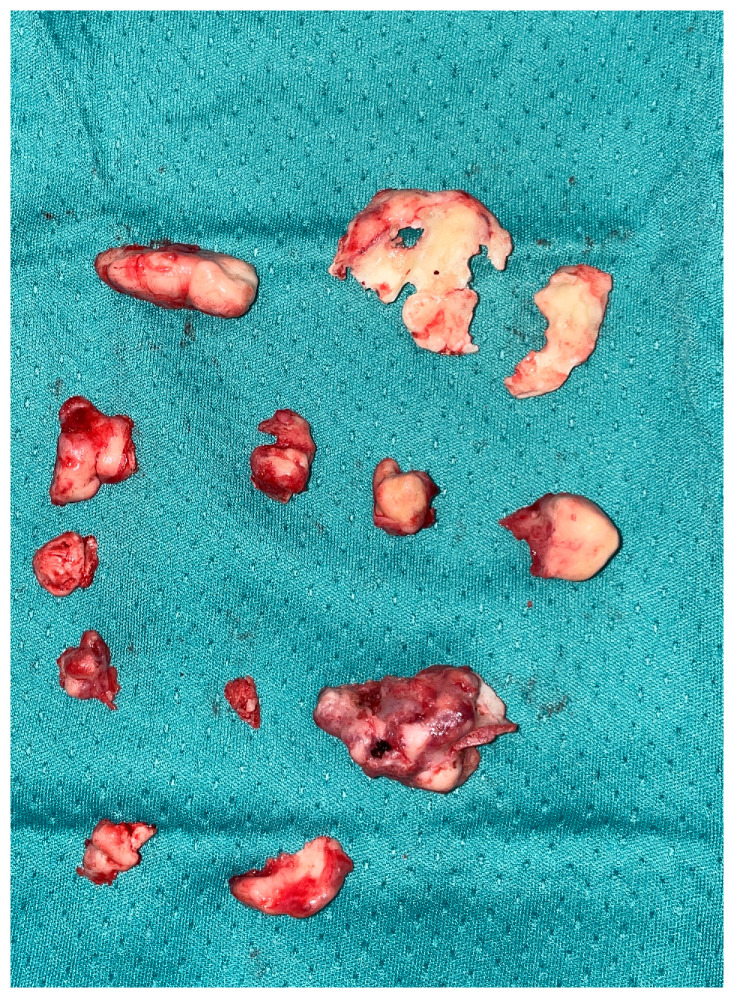
Intra-operative samples. All the fragments removed during the surgery are appreciated.

After this first phase of ‘open’ surgery, we moved on to the endoscopic phase by which the nasal cavities were explored bilaterally. On the right, the inferior turbinate appeared with a completely subverted structure invading the entire right nasal fossa; using a drill, the turbinate was detached and sent in its entirety for histological examination (Figure 5).

Then, using a dual approach, open from coronal and endoscopic access, the anterior wall of the middle skull base and the medial wall of the right orbit were exposed to completely remove the lesion with the neurosurgeon who removed the part of the lesion occupying the posterior crista galli (Video S1).

Finally, the pericranial flap previously harvested to isolate the middle cranial fossa from the nasal cavities was repositioned, the frontal bone fragment removed during the craniotomy was repositioned and fixed with plates and screws, and endoscopic control of the nasal cavities was performed to ensure accurate hemostasis. Two swabs were placed, one for each nasal fossa, and removed on the third day, and two drains at the level of the coronal surgical access were removed after 5 days.

**Figure 6:**
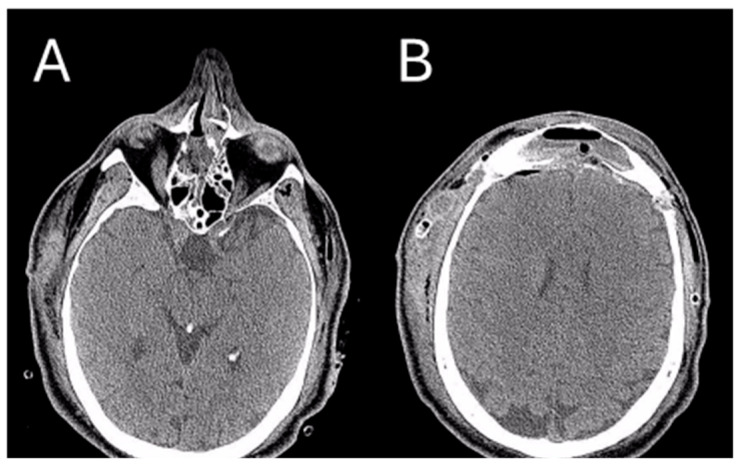
Post-operative CT image of Patient 2. Two axial cuts of the post-operative CT scan are appreciatedat the same level as those shown in Figure 3 to demonstrate the outcomes of the surgical removal. (**A**) the absence of the lesion localized at the ethmoidal level (**B**) The absence of the lesion localized in the right frontal sinus.

After the surgical intervention, a skull CT scan was performed, revealing a near-complete removal of the lesion (Figure 6).

**Figure 7:**
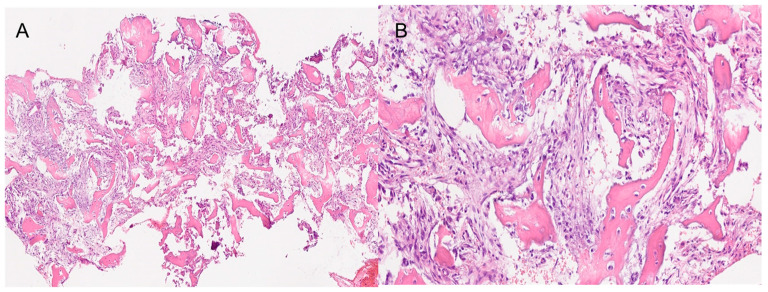
The histopathological images show a section of the bones samples. (**A**) 4× magnification. (**B**) 20× magnification. Both images show the fibrous component with variable, medium to low cellularity. Osteoblastic rimming is observed in all trabeculae. The osseous component exhibits amorphous crystalline, calcific deposits. Segments of respiratory-type epithelium with areas of squamous metaplasia are also observed.

The histological result of the sample sent in the second surgery was Cemento-Ossifying Fibroma, a surprising result as this type of ossifying fibroma of odontogenic origin usually affects the tooth-bearing region of the gnathic bones (Figure 7).

The patient continued follow-up at the maxillofacial surgery outpatient clinic with quarterly check-ups for one year following the intervention. An eye examination was performed six months after surgery and a new CT evaluation was scheduled one year after surgery. Throughout this period, no signs of worsening were reported.

## 3. Discussion

The term fibro-osseous lesion is a generic designation of a group of disorders characterized by the replacement of bone by benign cellular fibrous tissue containing foci of various degrees of mineralization [9,10]. The maxillofacial region is a highly affected area with a prevalence at the level of the frontal bone, paranasal sinuses, and maxillary bones [11].

In 2022, the WHO published an update a few years after the last classification of head and neck bone tumors, due to increasingly promising discoveries in molecular biology and genetics. Among the various pathologies described in the group of fibrous bone lesions, the most frequent are Fibrous Dysplasia (FD) and Ossifying Fibroma, currently to be classified under three specific nosological entities.

Fibrous dysplasia represents a sporadic benign skeletal anomaly, displaying a spectrum encompassing both the monostotic involvement of a single bone and polyostotic involvement of multiple bones. The latter manifestation may be associated with either McCune–Albright syndrome (MAS) or Jaffe–Lichtenstein syndrome (JLS). JLS is characterized by polyostotic FD and café-au-lait pigmented skin lesions, whereas MAS presents additional hyper-functional endocrinopathies, such as precocious puberty, hyperthyroidism, or acromegaly.

Gender distribution in FD is equal, with the monostotic form being more prevalent and primarily affecting individuals aged 20 to 30 years; on the other hand, polyostotic FD typically manifests in children younger than 10 years. While serum alkaline phosphatase (ALP) elevation may occur sporadically, levels of calcium, parathyroid hormone, 25-hydroxyvitamin D, and 1,25-dihydroxyvitamin D typically remain within normal ranges. In instances of extensive polyostotic FD, hypophosphatemia, hyperphosphaturia, and osteomalacia may manifest. Malignant transformation is infrequent. Clinical manifestations of FD encompass bone pain, pathological fractures, and bone deformities. Craniofacial bones are affected in approximately 10% of monostotic FD cases and 50% to 100% of polyostotic FD cases. The term craniofacial FD is employed when solely the cranial and facial bones are involved. Maxillary involvement is more common than mandibular, with a higher incidence observed in females [12]. Affected cranial or facial bones dictate the clinical features. Symptoms may encompass facial pain, headache, cranial asymmetry, facial deformity, tooth displacement, and visual or auditory impairment.

The radiographic characteristics exhibit a broad spectrum, dependent upon the balance between mineralized bone and fibrous tissue within the lesion. In early stages, FD lesions affecting craniofacial bones typically appear radiolucent, displaying either well-defined or poorly defined borders, and may present as either unilocular or multilocular formations. The progression of these lesions results in a transition to a mixed radiolucent/radiopaque appearance, with mature FD showcasing mottled radiopaque patterns often likened to ground glass, orange peel, or fingerprints. Borders of these lesions tend to be ill-defined, merging seamlessly with surrounding normal bone structures.

However, some typical features can be identified through various imaging modalities, with radiography (X-ray) providing an initial assessment and CT scans offering a detailed characterization of the lesion.

There is frequently an enlargement and deformation of the affected bone, resulting in a bulbous or fusiform shape. The lesion margins are typically well-defined, although they may appear somewhat hazy. The endosteal surface of the cortical bone may show scalloping due to the infiltration of fibrous tissue, and the cortex is often thinned, though it generally remains intact. Unlike other bone lesions, fibrous dysplasia typically does not elicit a periosteal reaction [13].

Histological characteristics of fibrous dysplasia include a fibroblastic proliferation within a collagenous stroma, which is typically bland and uniform and the presence of irregular trabeculae of immature, woven bone which are interspersed within the fibrous stroma. These trabeculae are often described as having a “Chinese character” or “alphabet soup” appearance and, typically, they are not lined by osteoblasts unlike many other bone lesions. Another characteristic is the presence of small, irregularly shaped islands of mature lamellar bone within the lesion [14].

A definitive cure for fibrous dysplasia remains elusive, and consensus on treatment protocols is lacking across the medical community. The spontaneous regression of FD is not observed. In cases where FD lesions are asymptomatic, stable, and devoid of deformities or functional limitations, a strategy of regular monitoring is recommended. Surgical intervention becomes imperative when vital structures are at risk of compression. Post-surgical recurrence rates of fibrous dysplastic lesions, especially in younger individuals and with less mature lesions, are notably high (approximately 50%). In cases where surgery is unsuitable, the alleviation of bone pain and inhibition of osteoclastic activity can be achieved through intravenous bisphosphonate therapy [15,16].

Cemento-Ossifying Fibroma (COF) represents a benign odontogenic tumor characterized by the presence of cementum within its structure. Its global prevalence stands at 3.1% of all oral tumors, with the adult variant typically manifesting in the third to fourth decade of life and the juvenile variant in the first decade. Proposed to originate from cells of the periodontal ligament, COF exhibits clinical features typified by asymptomatic swelling, slowly enlargement, being more prevalent in women, and primarily affecting the mandible with a predilection for the apical region of posterior teeth. Early signs may include teeth displacement, while teeth adjacent to the lesion often retain vitality. Consequently, patients may remain unaware of the lesion until it significantly alters their facial appearance. The tumor presents as a distinct entity from the surrounding bone and exhibits continuous growth, either gradual or rapid until surgical excision is performed [17].

The histological appearance reveals a dynamic blend of fibrous connective tissue stroma punctuated by areas of mineralization, exhibiting diverse morphologies often resembling cementum-like tissue. Within the stroma, a densely populated assembly of spindle-shaped fibroblastic cells was observed, with generally low vascularity. Noteworthy stromal features encompass the presence of multinucleated giant cells reminiscent of osteoclasts, appearing sporadically or clustering in focal aggregates. The mineralized component displayed an array of morphological configurations, encompassing trabecular and psammomatoid patterns. Trabeculae exhibited varying shapes and sizes, comprising thin or thick interconnecting strands, irregularly bulbous formations, and occasionally coalescing into extensive sheets, sometimes encircled by osteoblastic rimming. Likewise, psammoma-like bodies exhibited a spectrum of appearances, ranging from well-defined acellular spherical masses to less structured irregular masses, characterized by a basophilic core and peripheral hyalinization. Occasionally, these bodies manifested delicate, feathery outlines or fused into intricate ‘ginger root’ patterns [18].

Cemento-Ossifying Fibroma exhibits a radiographic presentation that evolves with its degree of mineralization. Initially, it appears radiolucent, with calcific flecks becoming more prominent as the lesion matures, eventually culminating in the formation of a fully radiopaque mass. Some studies suggest that a radiolucent pattern is more prevalent among younger individuals, while older patients tend to exhibit a mixed radiolucent-radiopaque appearance [19]. A defining characteristic of COF is its centrifugal growth pattern, resulting in a well-defined, round or oval mass expanding uniformly in all directions; additionally, the trabeculae display a mixed radiolucency-radiopacity, resembling a cotton wool appearance.

The inner aspect of the lesion can appear granular, similar to fibrous dysplasia, or exhibit a radiolucent periphery indicative of a fibrous capsule. There may also be cortical bone expansion and a mixture of radiolucent and radiopaque tissues [20].

Treatment strategies for Cemento-Ossifying Fibroma vary depending on the size and aggressiveness of the lesion. Curettage or enucleation is commonly recommended as the primary therapy for COF, facilitated by the well-defined borders of the lesion. In cases of recurrence or when the lesion displays aggressive behavior, radical resection followed by bone grafting may be considered. However, it is important to note that despite these treatment modalities, recurrence rates remain a concern. Studies have reported recurrence rates ranging from 28% to 30% in cases treated with surgical curettage or enucleation. Similarly, investigations by Slootweg and Muller have indicated comparable recurrence rates between conservative surgery and more extensive resection techniques [21]. Recurrence has been observed to manifest within a wide timeframe, occurring anywhere from six months to seven years post-surgery [22].

The overlap in diagnostic criteria among fibro-osseous lesions in the mandible or maxilla may lead to confusion among clinicians, radiologists, pathologists, and oral surgeons during diagnosis and treatment.

However, the differential diagnosis of these lesions should not be evaluated solely within the group of fibro-osseous lesions. Benign bone and cartilage tumors must also be considered, as they can present with similar characteristics and are also common in the head and neck region.

Osteoblastoma, whether intra-osseous or periosteal, occurs most commonly in the second–third decades and shows a slight female preponderance. Chondroblastoma in the head and neck region is typically located around the temporomandibular joint and the squamous part of the temporal bone. Chondromyxoid fibroma, a rare benign chondroid neoplasm with a zonal architecture composed of chondroid, myxoid, and myofibroblastic areas, can develop within or on the bone surface. In about 90% of tumors, the genetic driver event involves a mutation in the glutamate receptor gene, GRM1, which seems unique to chondromyxoid fibroma and is rare to absent in other cartilaginous tumors.

The desmoplastic fibroma of bone is a locally aggressive fibroblastic/myofibroblastic tumor composed of benign spindle cells embedded in a collagenous background, mimicking desmoid-type fibromatosis. In the jaw, 82% affect the mandible, and almost 70% are diagnosed before the age of 30 years. Clinically, desmoplastic fibroma is asymptomatic; however, swelling, facial asymmetry, pain, and trismus may be present. Radiologically, desmoplastic fibroma manifests as a well-defined radiolucency, either uni- or multi-locular. Phenotypically, spindle cells are primarily positive for vimentin and smooth muscle actin, with a low Ki67 proliferative marker. The recurrence rate after curettage is 31%; after enucleation it is 25% and after resection it is only 10% [23].

Accurate diagnosis holds significant importance in fibro-osseous lesions, given the array of available treatment options. Achieving a definitive diagnosis relies heavily on comprehensive evaluation encompassing demographic, clinical, and radiological data.

The radiological study should encompass not only CT imaging but often a first-level examination such as X-ray, which can be helpful and is often more readily available than tomography.

## 4. Conclusions

The updated classification of odontogenic and maxillofacial bone tumors by the World Health Organization (WHO) in 2022 has significantly impacted diagnostic and therapeutic approaches. Fibro-osseous lesions of the maxillofacial bones, including Fibrous Dysplasia (FD), Psammomatoid Ossifying Fibroma (PSOF), Juvenile Trabecular Ossifying Fibroma (JTOF), and other variants, present diagnostic challenges due to their histological similarities but distinct clinical manifestations. Precise differentiation is crucial for optimal patient management. Through the presentation of two cases, Fibrous Dysplasia and Cemento-Ossifying Fibroma, this article underscores the importance of a systematic approach in evaluating and treating fibro-osseous lesions. Despite their complexities, advancements in diagnostic techniques and treatment modalities offer hope for improving outcomes for patients affected by these conditions. Continued research and collaboration among clinicians, pathologists, and surgeons are essential to further enhance our understanding and management of fibro-osseous lesions in the maxillofacial region.

## Data Availability

The original contributions presented in the study are included in the article/Supplementary Materials, further inquiries can be directed to the corresponding author.

## Supplementary Materials

The following supporting information can be downloaded at: https://www.mdpi.com/article/10.3390/jcm13113233/s1, Video S1: Dual Maxillo and NCH Approach. A phase of lesion removal is appreciated with a dual approach, endoscopic from below and open neurosurgical from above.

## References

[1] Vered M., Wright J.M. (2022). Update from the 5th Edition of the World Health Organization Classification of Head and Neck Tumors: Odontogenic and Maxillofacial Bone Tumours. Head Neck Pathol..

[2] Nelson B.L., Phillips B.J. (2019). Benign Fibro-Osseous Lesions of the Head and Neck. Head Neck Pathol..

[3] Saló Bru G., Sociedad Espanola de Reumatología (2010). Fibrous dysplasia. Chondrodysplasias. Manual de Enfermedades Óseas.

[4] Chattopadhyay A., Jain S., Sharma A. (2020). Craniofacial Fibrous Dysplasia. J. Clin. Rheumatol..

[5] Resnick C.M., Novelline R.A. (2008). Cemento-osseous dysplasia, a radiological mimic of periapical dental abscess. Emerg. Radiol..

[6] Cascino F., Aboh I.V., Giovannoni M.E., Pini N., Zerini F., Del Frate R., Carangelo B.R., Xu J., Gabriele G., Gennaro P. (2021). Orthognathic surgery: A randomized study comparing Piezosurgery and Saw techniques. Ann. Ital. Chir..

[7] Massarelli O., Vaira L.A., De Riu G. (2022). A new aesthetic pretrichial approach for upper third-facial fractures and pathologies: The “Crown incision”. J. Plast. Reconstr. Aesthetic Surg..

[8] Shohet M.R., Laedrach K., Guzman R., Raveh J. (2008). Advances in approaches to the cranial base: Minimizing morbidity. Facial Plast. Surg..

[9] Prabhu S., Sharanya S., Naik P.M., Reddy A., Patil V., Pandey S., Mishra A., Rekha K. (2013). Fibro-osseous lesions of the oral and maxillo-facial region: Retrospective analysis for 20 years. J. Oral Maxillofac. Pathol..

[10] MacDonald-Jankowski D.S. (2004). Fibro-osseous lesions of the face and jaws. Clin. Radiol..

[11] Lentzen M.P., Riekert M., Grozinger P., Zirk M., Nickenig H.J., Zöller J.E., Kreppel M. (2021). Anatomical and volumetric analysis of fibro-osseous lesions of the craniofacial skeleton. J. Craniomaxillofac. Surg..

[12] AlAmmari G., Almahbub A., Alfawwaz F. (2023). Navigating Fibrous Dysplasia of the Middle Turbinate: A Decade-Long Journey and Comprehensive Management Strategies. Cureus.

[13] Alsufyani N., Alzahrani A. (2024). Imaging of Fibro-osseous Lesions and Other Bone Conditions of the Jaws. Dent. Clin. N. Am..

[14] Parman L.M., Murphey M.D. (2000). Alphabet soup: Cystic lesions of bone. Semin. Musculoskelet. Radiol..

[15] Feller L., Wood N.H., Khammissa R.A., Lemmer J., Raubenheimer E.J. (2009). The nature of fibrous dysplasia. Head Face Med..

[16] Chapurlat R. (2007). Medical therapy in adults with fibrous dysplasia of bone. J. Bone Miner. Res..

[17] Katti G., Khan M.M., Chaubey S.S., Amena M. (2016). Cemento-ossifying fibroma of the jaw. BMJ Case Rep..

[18] Collins L.H.C., Zegalie N.F.T., Sassoon I., Speight P.M. (2023). A Clinical, Radiological and Histopathological Review of 74 Ossifying Fibromas. Head Neck Pathol..

[19] Jayachandran S., Sachdeva S. (2010). Cemento-ossifying fibroma of mandible: Report of two cases. J. Indian Acad. Oral Med. Radiol..

[20] Chidzonga M., Sunhwa E., Makunike-Mutasa R. (2023). Ossifying Fibroma in the Maxilla and Mandible: A Case Report with a Brief Literature Review. Cureus.

[21] Slootweg P.J., Müller H. (1990). Juvenile ossifying fibroma. Report of four cases. J. Craniomaxillofac. Surg..

[22] Divyadharshini V., Jayanth K., Ganapathy D. (2023). A Painless Bump: A Case Report of Cemento-Ossifying Fibroma of the Anterior Maxilla. Cureus.

[23] Gomez-Pena S., Rueda de Eusebio Á., Arrazola García J., Romero Fernández P., Moreno Casado M.J., Crespo Rodríguez A.M. (2024). Update of cartilaginous tumours according to the WHO classification 2020. Radiologia.

